# Altered Body Composition and Increased Resting Metabolic Rate Associated with the Postural Instability/Gait Difficulty Parkinson's Disease Subtype

**DOI:** 10.1155/2020/8060259

**Published:** 2020-03-17

**Authors:** Giovana Femat-Roldán, María Andrea Gaitán Palau, Inma Castilla-Cortázar, Georgina Elizondo Ochoa, Nancy Guadalupe Moreno, Irene Martín-Estal, Miguel Jiménez Yarza

**Affiliations:** ^1^Neurocenter, Monterrey, Nuevo Leon, Mexico; ^2^Tecnologico de Monterrey, Escuela de Medicina y Ciencias de la Salud, Ave. Morones Prieto 3000, Monterrey 64710, NL, Mexico; ^3^Fundación de Investigación HM Hospitales, Madrid, Spain

## Abstract

**Background:**

Weight loss in Parkinson's disease (PD) patients is a common but poorly understood manifestation. Several studies have reported that weight changes could be related to motor symptoms, drug side effects, dysphagia, depression, and/or dementia. Weight loss in PD is not a benign phenomenon and it has several clinical and prognostic implications with increased morbidity and mortality. Thus, it is crucial to determine nutritional changes in PD patients in order to prevent malnutrition and improve their quality of life.

**Objective:**

To compare body composition and resting metabolic rates between PD patients and controls.

**Methods:**

A total of 64 PD patients and 52 controls were studied. The Hoehn-Yahr scale was used to determine the disease stage, clinical and epidemiological data were recorded from verbal questionnaire, Inbody S10® was used to collect corporal parameters, and FitMate system was used to assess the resting metabolic rate.

**Results:**

No significant differences were found between both experimental groups in age, gender, height, cholesterol levels, and the presence of hypertension, diabetes, and hypo/hyperthyroidism. However, the PD group showed lower body fat mass, whole-body fat percentage, and greater resting metabolic rate compared to controls (*p* < 0.05), with no significant differences in musculoskeletal mass. Parkinson's disease postural instability/gait difficulty (PD-PIGD) subtype showed lower body fat parameters, increased fat-free mass, and higher resting metabolic rates.

**Conclusions:**

These results suggest that PD patients present an increased resting metabolic rate associated with the postural instability/gait difficulty PD subtype, allowing a selective decrease of body fat mass and not musculoskeletal mass. Of note, several disease-related factors may contribute to this weight loss in PD patients, being a complex and multifactorial consequence. Our findings could likely be one of the many contributing factors. However, present findings may further add to our understanding of the phenomenon of weight loss in patients with PD.

## 1. Introduction

Worldwide, Parkinson's disease (PD) is the second most common neurodegenerative disorder after Alzheimer's disease. Its incidence increases with age and affects approximately 1–4% of the population over 60 and 80 years, respectively [1]. PD is characterized by a progressive motor deficit, initially manifested by tremor and bradykinesia.

Unintentional weight loss is commonly seen in PD patients [2–11]. The prevalence and risk of malnutrition are approximately 24% and 60%, respectively [12]. Several disease-related factors may contribute to this weight loss observed in PD patients including nonmotor symptoms such as hyposmia [13], dysphagia, gastrointestinal dysfunction, depression, and dementia [8, 14], as well as motor impairments with hand-mouth incoordination and chewing difficulties, all leading to a reduced food intake [3].

Additionally, an increase in energy requirements in PD has been described due to muscular rigidity, tremor, and dyskinesia [8, 15, 16]. Resting metabolic rate (RMR), also called resting energy expenditure, is defined as the energy required by the body in a resting condition [17]. This energy requirement is important because it typically accounts for the largest portion of total energy needs.

Furthermore, other disease-unrelated factors might contribute to the aforementioned weight loss including aging [2, 6, 18], patient's socioeconomic status, dietary habits, and medication side effects [2, 4]. On the other hand, obesity and weight gain have also been described in the first stages of PD due to the reduced activity and drug adverse effects [19–22].

Although weight loss has been associated with PD progression, severity [10, 18, 20, 23–25], higher levodopa equivalent daily dose [2, 4], greater unified Parkinson's disease rating scale III score (UPDRS) [6, 24], and disease duration [6], it can occur in early phases of the disease [7, 26], in spite of having an adequate or even an increased energy intake [10, 26, 27].

Frequently, malnutrition and underweight are negatively associated with quality life in PD patients [2, 6, 13, 28, 29]. Both have been associated with vitamin D deficiency, reduced bone mineral density, bone fractures [3, 30, 31], cognitive decline [32], and dyskinesia [13, 28], being a major source of increased disability and mortality [13, 28].

Body composition measurements are objective methods for nutritional assessment in order to better understand weight changes in PD patients. Notwithstanding the increasing evidence regarding weight changes in PD, its causes are still not well understood, and body composition characteristics of PD patients are not yet well established. Some studies suggest that body composition changes in PD patients are due to the loss of body fat tissue [33], preserving musculoskeletal mass [34], while others propose that this weight loss could be related to the redistribution of muscle to fat [21]. In this fashion, an excess of adiposity associated with a lean body mass depletion (sarcopenic obesity) has been described in advanced stages of the disease [21, 22, 35].

For these reasons, it is critical to better understand the changes in body composition (e.g., fat and muscle mass) and metabolism that occur in PD patients in order to provide further insight on strategies to prevent these complications. In the present study, we aimed to compare body composition and resting metabolic rates of PD patients.

## 2. Materials and Methods

### 2.1. Subjects and Study Design

An observational case-control study of 64 PD patients and 52 controls was conducted from May 2016 to December 2017 at a private neurological care clinic. All patients with PD diagnosis who were consecutively seen in our clinic were selected to participate in the study. All the procedures were approved by the Bioethical Committee from our institution. All PD patients and controls signed a written informed consent prior to the participation in the study.

The following inclusion criteria for PD patients were used in the present study: age 30 years or older, written informed consent, and diagnosis of PD previously made by their primary care physician based on the Queen Square Bank Criteria. Similarly, the following exclusion criteria for PD subjects were included: clinically significant gastrointestinal, cardiovascular, hepatic, renal, hematological, neoplastic, endocrine, immunodeficiency, pulmonary, or other disorder or disease, metal implants, pacemaker, and deep brain stimulation surgery.

Age and gender matched controls were recruited among clinic visitors. Age 30 years or older and written informed consent were the inclusion criteria employed. Correspondingly, clinically significant gastrointestinal, cardiovascular, hepatic, renal, hematological, neoplastic, endocrine, immunodeficiency, pulmonary, or other disorder or disease, metal implants, and/or pacemaker were the exclusion criteria included in control subjects.

All PD patients were treated with levodopa at the time of the assessment. Doses ranged from 250 to 1250 mg/day. Complete data of other pharmacological treatments were missing.

Weights from PD patients and controls were measured in a calibrated balance beam scale (Seca 700, Mundo Medico, Mexico) with 12 hours of fasting, without shoes, and with minimal clothes. Height was measured with telescopic height rod without shoes, standing in erect position. Clinical and epidemiological data including age, sex, duration of disease, Hoehn-Yahr stage, and UPDRS score were recorded. Patient examination and assessment were performed according to standard protocols by a board certified neurologist who had received training in the use of UPDRS [36].

Body mass index (BMI) was calculated using the formula weight (kg)/height^2^ (m). A questionnaire was used in order to recall the average weight between ages 30 and 40 in patients and controls. These results allow us to calculate their self-reported weight using the formula: usual weight − actual weight.

For a subanalysis, PD patients were further divided into two groups based on their predominant symptoms. For each patient, the mean UPDRS tremor score (average of part II, Item 2.16, and part III, Items 3.20–3.21) and the mean UPDRS PD-PIGD score (average of part II, Items 2.13–2.15, and part III, items 3.29 and 3.30) were obtained as reported in previous studies [23, 37].

Based on the ratio of TD score to PIGD score (subtype ratio), PD patients were divided into two groups PD-tremor dominant and indeterminate group (PD-TD + indeterminate) or PD postural instability/gait difficulty group (PD-PIGD). PD-TD + indeterminate group if the ratio was >1.0 (*n* = 25) or into the PD-PIGD if the ratio was <1.0 (*n* = 39) [23].

Bioelectrical impedance analysis (BIA) data collection of the 116 participating subjects was done using the Inbody S10® system (InBody, USA). The documented parameters were weight, lean mass (LM), fat-free mass (FFM), body fat mass (BFM), musculoskeletal mass (MSM), body mass index (BMI), body fat percentage (%BF), intracellular water (ICW), extracellular water (ECW), basal metabolic rate (BMR), waist circumference (WC), visceral fat area (VFA), bone mineral content (BMC), body cell mass (BCM), arm circumference (AC), and protein and mineral levels. All subjects were positioned in supine position. Contact electrodes were placed in thumbs and middle fingers of both hands and in the space between malleolus and heel of lower extremities, according to previous studies [21, 35].

Resting metabolic rate (RMR) was measured during off-state in all PD patients using the Fitmate MED® indirect calorimetry system (Cosmed, Italy), after 12-hour fasting. In this way, all PD patients were measured without the medication effect that has shown to reduce energy expenditure [38]. Patients were placed in supine position wearing an RMR mask during an initial habituation phase (5 minutes), which was followed by 10 minutes of continuous data acquisition. The Fitmate MED® indirect calorimetry system uses standard metabolic formulas to calculate oxygen uptake and monitor several parameters related to RMR, for example, oxygen uptake (VO_2_), ventilation (Ve), respiratory frequency (RF), heart rate (HR), and O_2_ expired fraction (FeO_2_). RMR (kcal/day) was estimated by a modified Weir equation: RMR = [5.675 × VO_2_ + 1.593 × VCO_2_ − 21.7], where VO_2_ is volume of oxygen in breath (ml/min) and VCO_2_ is carbon dioxide output (ml/min) [39].

### 2.2. Statistical Analysis

Demographic, clinical, RMR, and BIA parameters were introduced in a database using Microsoft Excel spreadsheet (16.0.6568.2036 version). SPSS 22 (Statistical Package for Social Sciences, IBM Statistics) was used for statistical analysis. Descriptive statistics were used to characterize the subjects. *χ*^2^ square and Student's *t*-tests were used to compare qualitative and quantitative variables, respectively. Differences were considered significant at a level of *p* < 0.05.

## 3. Results

In the present study, 64 patients and 52 age and gender matched controls were recruited. Clinical and demographical characteristics of these study subjects are summarized in Table 1. No significant differences were found in age, gender, and past medical history (presence of hypertension, diabetes mellitus and thyroid disease) between both experimental groups (Table 1). Similarly, no significant differences were observed between PD patients and controls in usual and actual weight. However, statistical disparities were found in the self-reported weight change, having PD patients self-reported less weight gain (1.4 kg) than control subjects (10.3 kg) (*p*=0.001) (Table 1). Approximately 37.5% of PD patients stated a decrease in their self-reported weight (between 1 and 33 kg) compared to the 13.5% reported by control subjects (between 1.5 and 12.5 kg).

Regarding all body composition parameters measured in the present study, significant differences were found in body fat mass (*p*=0.007), body fat percentage (*p*=0.024), visceral fat area (*p* ≤ 0.001), waist circumference (*p* ≤ 0.001), arm circumference (*p*=0.007), and right and leg weight proportions (*p* ≤ 0.001) in PD patients compared to controls (Table 1). All these parameters were lower in PD patients, except for right and left leg weight proportions, both being higher in PD patients. No significant differences were observed in musculoskeletal mass between both groups. A subanalysis did not show statistical differences in the variables previously described.

Based on the ratio of tremor score to postural instability/gait difficulty score (subtype ratio), PD patients were divided into two groups depending on the predominant symptoms: the TD + indeterminate group (*n* = 25) and the PD-PIGD group (*n* = 39) (Table 2). No significant differences were found in age, gender, and past medical history (presence of hypertension, diabetes mellitus, and thyroid disease), levodopa daily dose, presence and duration of dyskinesias between both groups. Additionally UPDRS parts I and IV showed no significant differences between groups. However, UPDRS parts II and III were significantly higher in the PD-PIGD group. Significant differences were found in several BIA and clinical parameters. The PD-PIGD group exhibited lower body fat mass (*p*=0.020), body fat percentage (*p*=0.001), waist circumference (*p*=0.014), and visceral fat area (*p*=0.029) compared to the TD + indeterminate group. Conversely, PD-PIGD patients exhibited higher fat-free mass (*p*=0.049) compared to TD + indeterminate group (Table 2).

RMR analysis was performed in 26 PD patients and 29 controls. Both groups had no significant differences in age, gender, weight, and height. PD patients presented an increase in RMR (*p*=0.010) and RMR per kilogram of total body weight (*p*=0.002) compared to controls (Table 3). Furthermore, PD patients with measured RMR rates were divided into two groups depending on the predominant symptoms: the TD + indeterminate group (*n* = 14) and the PD-PIGD group (*n* = 12) (Table 4). Both groups had no significant differences in age, gender, weight, and height. Nevertheless, an increase in RMR (*p*=0.001) and RMR per kilogram of total body weight (*p*=0.010) was observed in the PD-PIGD group compared to the TD + indeterminate group (Table 4).

**Figure 1:**
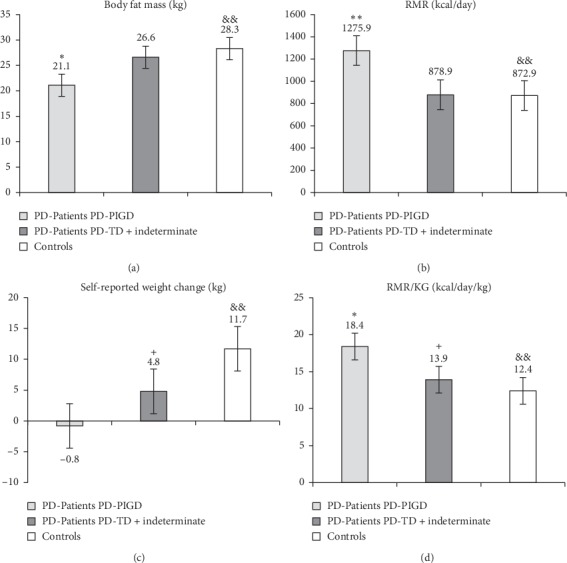
Body fat mass (a), resting metabolic rate (RMR) (b), self-reported weight change (c), and RMR per kg of total body weight (d), in PD patients and controls. ^*∗*^*p* < 0.05 PD-PIGD (PD1) versus PD-TD + indeterminate (PD2); ^*∗∗*^*p* ≤ 0.001 PD-PIGD (PD1) versus PD-TD + indeterminate (PD2); ^&^*p* < 0.05 PD-PIGD (PD1) versus controls; ^&&^*p* ≤ 0.001 PD-PIGD (PD1) versus controls; ^+^*p* < 0.05 PD-TD + indeterminate (PD2) versus controls. PD patients: Parkinson's disease patients; PD-PIGD: Parkinson's disease postural instability/gait difficulty; PD-TD + indeterminate: Parkinson's disease tremor dominant; RMR: resting metabolic rate; RMR/KG: resting metabolic rate per kilogram of total body weight; Self-reported weight change: calculated as the difference between actual weight and usual weight.

The comparison between TD + indeterminate group, PD-PIGD, and controls is shown in Figure 1. Body fat mass was significantly lower in PD patients compared to controls, showing the PI-PIGD group a greater difference (*p*=0.001). In contrast, PD patients exhibited resting metabolic rates (both RMR and RMR per kilogram of body weight) higher than controls, showing also the PD-PIGD group with the greatest difference (*p*=0.000). Finally, the comparison of the self-reported weight change showed significant differences between both PD groups (PD-PIGD and TD + indeterminate) and controls (*p*=0.000 and *p*=0.043, respectively) and also directly correlated with body fat mass, being inversely proportional to basal metabolic rate. These results hint that PD-PIGD patients exhibit an increase in RMR that could allow a selective decrease of body fat mass and not musculoskeletal mass.

## 4. Discussion

In the present study, demographic, BIA parameters of body composition and RMR were measured and compared between PD patients and age and gender matched controls. In addition, these parameters were compared between PD patients with tremor or postural instability/gait difficulty predominant symptoms.

In our subjects, patients with postural instability/gait difficulty (PIDG) were more prevalent than those who were tremor-dominant or indeterminate (39 versus 25, respectively). This is not the usual distribution of PD subtypes seen in most movement disorders clinics. We considered such distribution because one of the main strengths of our clinic is our neurorehabilitation program. Numerous patients are referred from other movement disorders clinics for rehabilitation, requiring for PD-PIDG patients more rehabilitation than those with tremor, showing our population this distribution.

Overall, the weight difference between PD patients and control subjects was minimal and had no statistical significance, opposed to findings observed previously that described that overweight is more prevalent in PD patients than in healthy subjects in Mexico [19] and other countries [22].

Self-reported weight loss was more common in PD than in age and gender matched controls, a result consistent with previous observations [2,5–11].

Lower body fat parameters were observed in PD patients, including body fat mass and body fat percentage, as well as waist and arm circumferences. However, no significant differences were observed in musculoskeletal mass. This data does not support the sarcopenic obesity theory proposed previously in PD [21], where PD patients exhibit greater body fat mass and a decrease in muscle and bone masses. Recently similar findings were reported [33], assuming that this could be due to inflammation, hyperinsulinemia, or increased sympathetic activity. Regardless that the unintentional weight loss is stated in approximately 37.5% of PD patients, the musculoskeletal mass did not show statistical differences between PD patients and control subjects and was slightly higher in PD patients (26.2 kg) than in controls (25.5 kg). Considering the statistically significant difference between both experimental groups in body fat mass but not in musculoskeletal mass, these results suggest that the self-reported weight change in PD patients could be a consequence of the body fat mass loss with the retention of fat-free mass. Of note, several disease-related factors may contribute to this weight loss in patients with PD, being a complex and multifactorial consequence. However, the present findings may further add to our understanding of the phenomenon of weight loss in patients with PD.

In the present study, an increased RMR was also found in PD patients compared to controls, suggesting that an augmented RMR may be an inherent feature of the disease as suggested by previous studies [15, 16].

When PD patients were divided and compared into two groups depending on the predominant symptoms (TD + indeterminate group versus PD-PIGD group), UPDRS parts II and III were significantly higher in the PD-PIGD group, being consistent with previously reported studies where the PD-PIGD subtype showed a more severe form of the disease [40]. The PD-PIGD group showed lower body fat parameters, increased fat-free mass, and higher resting metabolic rates. These results suggest that such altered body composition and increased resting metabolic rate is more strongly associated with the postural instability/gait difficulty PD subtype than the tremor dominant or indeterminates. However, these findings have not been described in our population previously.

It is important to notice that the present study has several limitations: the use of BIA and not the more accurate dual-energy X-ray absorptiometry (DEXA) for the assessment of body composition, the cross-sectional design, a small sample size, the unusual distribution of PD subtypes, the limitation of recalled usual weight, and the missing data about other pharmacological treatments and the Mini Mental State Examination of our patients confines our ability to draw conclusions about causality; but these results may further add to our understanding the phenomenon of weight loss in PD patients and may hint that the altered body composition observed in these patients may be influenced by an increased energy expenditure and not just by a reduced energy intake. Also, these data suggest that muscle rigidity, observed in postural instability/gait difficulty PD subtype, might be more strongly associated to this increase in energy expenditure than involuntary movements such as tremor.

## 5. Conclusions

Weight loss and malnutrition, two of the nonmotor signs frequently observed in PD, are negatively associated with quality of life. Both alterations seem to be the result of a negative energy balance where energy expenditure exceeds energy intake, leading to body fat loss and malnutrition.

In this regard, results in the present study suggest that PD patients present increased resting metabolic rate associated specially with the postural instability/gait difficulty PD subtype. This could allow a selective decrease of body fat mass and not musculoskeletal mass. Finally, weight loss in PD patients is probably a complex multifactorial consequence and our findings are likely one of the many contributing factors.

Nonetheless, future studies with larger sample sizes are needed to advance in the comprehension of altered body composition and resting energy expenditure in PD patients in order to design strategies to prevent weight loss and malnutrition.

## Figures and Tables

**Table 1:** Demographic and BIA parameters of PD patients and control subjects.

Variables	PD patients (*n* = 64)		Controls (*n* = 52)		*p* value
	Mean	SD or %	Mean	SD or %	
Age (years)	67	±12	64	±12	0.107
Gender					
Male	31	48.4%	29	55.8%	0.549
Female	33	51.6%	23	44.2.0%	
Hypertension (*n*)	27	42.2%	16	30.8%	0.283
Diabetes mellitus (*n*)	9	14.1%	11	21.2%	0.448
Thyroid disease (*n*)	7	10.9%	7	13.5%	0.898
Usual weight (kg)	69	±14.1	65	±9.73	0.053
Actual weight (kg)	70.7	±16.1	74.9	±15.0	0.156
**Self-reported weight change (kg)**	**1.4**	±**16.2**	**10.3**	±**11.7**	**0.001**
Height (cm)	162.0	±11.4	164.2	±8.3	0.252
Proteins (kg)	9.1	±2.4	10.7	±11.7	0.276
Lean mass (kg)	44.1	±11.8	43.8	±9.7	0.920
Minerals (kg)	3.4	±0.7	3.2	±0.7	0.401
Fat-free mass (kg)	47.3	±11.8	46.9	±9.8	0.859
**Body fat mass (kg)**	**23.2**	±**9.5**	**28.3**	±**10.2**	**0.007**
Musculoskeletal mass (kg)	26.2	±8.7	25.5	±6.0	0.614
BMI (kg/m^2^)	26.9	±4.6	27.8	±4.9	0.296
**Body fat percentage (%)**	**32.5**	±**10.0**	**36.6**	±**8.8**	**0.024**
Intracellular water (L)	21.1	±5.5	21.1	±4.6	0.991
Extracellular water (L)	13.6	±3.3	13.4	±2.6	0.686
Basal metabolic rate (kcal/day)	1,390.1	±254.9	1,382.9	±210.6	0.869
**Waist circumference (cm)**	**81.3**	**±11.7**	**92.2**	**±13.5**	**≤0.001**
**Visceral fat area (cm** ^**2**^ **)**	**102.5**	**±40.8**	**130.6**	**±36.1**	**≤0.001**
Bone mineral content (kg)	2.8	±0.7	2.7	±0.5	0.975
Body cell mass (kg)	29.7	±8.6	30.7	±7.2	0.485
**Arm circumference (cm)**	**29.9**	±**4.4**	**32.1**	±**4.1**	**0.007**
Right leg (kg)	6.2	±1.9	5.8	±1.4	0.227
**Right leg weight proportion (kg)**	**104.0**	±**16.9**	**93.3**	±**10.4**	**≤0.001**
Left leg (kg)	7.9	±2.5	7.5	±1.8	0.388
**Left leg weight proportion (kg)**	**103.5**	±**16.6**	**93.7**	±**9.7**	**≤0.001**
PD history and severity					
Disease duration (years)	8.5	±6.4			
Age at onset (years)	55.6	±6.3			
Hoehn and Yahr stage					
Stage I (*n*)	5	8.1%			
Stage II (*n*)	9	14.5%			
Stage III (*n*)	28	45.2%			
Stage IV (*n*)	28	29.0%			
Stage V (*n*)	2	3.2%			
UPDRS part II	18.3	±8.8			
UPDRS part III	31.1	±15.4			
Levodopa daily dose (mg/day)	482.8	±432.8			

BMI: body mass index. All values are expressed as mean ± SD or as absolute numbers and percentage. The statistics used were *t*-test and *χ*^2^ test.

**Table 2:** Demographic and BIA parameters of PD-T + indeterminate and PD-PIGD patients.

Variables	PD-TD + indeterminate (*n* *=* *25*)		PD-PIGD (*n* *=* *39*)		*p* value
	Mean	SD or %	Mean	SD or %	
Age (years)	65	±11	69	±13	0.175
Gender					
Male	9	36.0%	22	56.4%	0.181
Female	16	64.0%	17	43.6%	
Evolution (years)	9	5.0%	8	7.0%	0.386
Hypertension (*n*)	11	44.0%	16	41.0%	0.814
Diabetes mellitus (*n*)	4	16.0%	5	12.8%	0.721
Thyroid disease (*n*)	3	12.0%	4	10.3%	0.827
Usual weight (kg)	66	±13.1	71	±14.4	0.125
Actual weight (kg)	70.6	±15.7	70.8	±16.5	0.961
Self-reported weight change (kg)	4.8	±15.7	−0.8	±16.4	0.181
Height (cm)	159.5	±11.9	163.7	±11.0	0.152
Proteins (kg)	8.4	±2.1	9.5	±2.5	0.078
Lean mass (kg)	41.0	±9.5	46.0	±12.7	0.095
**Minerals (kg)**	**3.1**	±**0.7**	**3.5**	±**0.7**	**0.021**
**Fat-free Mass (kg)**	**43.6**	±**10.1**	**49.6**	±**12.4**	**0.049**
**Body fat mass (kg)**	**26.6**	±**10.1**	**21.1**	±**8.5**	**0.020**
Musculoskeletal mass (kg)	23.6	±6.1	27.9	±9.8	0.057
BMI (kg/m^2^)	28.1	±5.2	26.1	±4.1	0.092
**Body fat percentage (%)**	**37.6**	±**9.5**	**29.2**	±**9.1**	**0.001**
Intracellular water (L)	19.5	±4.5	22.1	±5.8	0.054
**Extracellular water (L)**	**12.5**	±**2.9**	**14.3**	±**3.4**	**0.031**
**Basal metabolic rate (kcal/day)**	**1,311.4**	±**217.2**	**1,440.6**	±**266.8**	**0.047**
**Waist circumference (cm)**	**85.8**	±**11.2**	**78.5**	±**11.3**	**0.014**
**Visceral fat area (cm** ^**2**^ **)**	**121.0**	±**41.5**	**90.6**	±**36.2**	**0.003**
Bone mineral content (kg)	2.6	±0.7	2.8	±0.7	0.177
Body cell mass (kg)	27.7	±6.6	30.9	±9.5	0.146
Arm circumference (cm)	30.5	±5.4	29.5	±3.6	0.390
**Right leg (kg)**	**5.4**	±**1.6**	**6.7**	±**2.0**	**0.007**
**Right leg weight proportion (kg)**	**94.3**	±**15.2**	**110.2**	±**15.0**	**≤0.001**
**Left leg (kg)**	**6.8**	±**2.1**	**8.6**	±**2.5**	**0.004**
**Left leg weight proportion (kg)**	**94.2**	±**15.5**	**109.5**	±**14.6**	**≤0.001**
UPDRS-I	3.8	±2.0	3.9	±2.8	0.060
**UPDRS-II**	**16.2**	**±5.9**	**19.4**	**±9.6**	**0.028**
**UPDRS-III**	**26.48**	**±10.24**	**34.05**	**±17.42**	**0.037**
UPDRS-IV	4.48	±3.16	4.78	±3.00	0.504
Dyskinesias	5	20%	6	15.4%	0.228
None (*n*)	20	80%	33	84.6%	
1–25% (*n*)	1	4%	3	7.7%	
26–50% (*n*)	2	8%	2	5.1%	
51–75% (*n*)	2	8%	0	0%	
76–100% (*n*)	0	0%	1	2.6%	
Wearing off	20	80%	29	74.4%	0.270
None (*n*)	6	24%	9	23.1%	
1–25% (*n*)	6	24%	10	25.6%	
26–50% (*n*)	7	28%	6	15.4%	
51–75% (*n*)	4	16%	9	23.1%	
76–100% (*n*)	2	8%	5	12.8%	
Levodopa daily dose (mg/day)	542.00	±465.63	444.87	±412.16	0.296

BMI: body mass index; PD-TD + indeterminate: Parkinson's disease tremor dominant and indeterminate; PD-PIGD: Parkinson's disease postural instability/gait difficulty. All values are expressed as mean ± SD or as absolute numbers and percentage. The statistics used were *t*-test and *χ*^2^ test.

**Table 3:** RMR of PD patients and control subjects.

Variables	PD patients (*n* *=* 26)		Controls (*n* = 29)		*p* value
	Mean	SD or %	Mean	SD or %	
Age (years)	67.3	±12.3	63.6	±11.8	0.105
Gender					
Male	13	50%	9	31%	0.247
Female	13	50%	20	69%	
Weight (kg)	67.9	±13.4	72.5	±13.9	0.793
Height (cm)	163.0	±13.4	162.1	±8.4	0.214
**RMR (kcal/day)**	**1062.1**	**±330.0**	**872.9**	**±184.3**	**0.010**
**RMR/KG (kcal/day/kg)**	**16.0**	**±4.6**	**12.4**	**±3.4**	**0.002**

RMR: resting metabolic rate; RMR/KG: resting metabolic rate per kilogram of total body weight. All values are expressed as mean ± SD or as absolute numbers and percentage. The statistics used were *t*-test and *χ*^2^ test.

**Table 4:** RMR of PD-TD + indeterminate and PD-PIGD patients.

Variables	PD-PIGD (*n* = 12)		PD-TD + indeterminate (*n* = 14)		*p* value
	Mean	SD or %	Mean	SD or %	
Age (years)	64.3	±12.3	67.2	±13.7	0.547
Gender					
Male	7	58%	6	43%	0.619
Female	5	42%	8	57%	
Weight (kg)	69.9	±12.9	66.1	±14.0	0.475
Height (cm)	167.6	±10.6	158.9	±14.6	0.102
**RMR (kcal/day)**	**1275.9**	**±287.0**	**878.9**	**±247.8**	**0.001**
**RMR/KG (kcal/day/kg)**	**18.4**	**±3.1**	**13.9**	**±4.8**	**0.010**

PD-TD + indeterminate: Parkinson's disease tremor dominant and indeterminate; PD-PIGD: Parkinson's disease postural instability/gait difficulty; RMR: resting metabolic rate; RMR/KG: resting metabolic rate per kilogram of total body weight. All values are expressed as mean ± SD or as absolute numbers and percentage. The statistics used were t-test and *χ*^2^ test.

## Data Availability

The data used to support the findings of this study are available from the corresponding author upon request.

## Abbreviations

AC: Arm circumference
BCM: Body cell mass
%BF: Body fat percentage
BFM: Body fat mass
BIA: Bioelectrical impedance analysis
BMC: Bone mineral content
BMI: Body mass index
BMR: Basal metabolic rate
ECW: Extracellular water
FeO_2_: O_2_ expired fraction
HR: Heart rate
ICW: Intracellular water
LM: Lean mass
FFM: Fat-free Mass
MSM: Musculoskeletal mass
PD: Parkinson's disease
PD-TD + indeterminate group: Parkinson's disease tremor dominant + Parkinson's disease indeterminate group
PD-PIGD: Parkinson's disease postural instability/gait difficulty
RF: Respiratory frequency
RMR: Resting metabolic rate
UPDRS: Unified Parkinson's disease rating scale
VFA: Visceral fat area
Ve: Ventilation
VO_2_: Oxygen uptake
WC: Waist circumference.
